# The *priB* Gene of *Klebsiella pneumoniae* Encodes a 104-Amino Acid Protein That Is Similar in Structure and Function to *Escherichia coli* PriB

**DOI:** 10.1371/journal.pone.0024494

**Published:** 2011-09-08

**Authors:** Linda Berg, Matthew E. Lopper

**Affiliations:** Department of Chemistry, University of Dayton, Dayton, Ohio, United States of America; Tulane University Health Sciences Center, United States of America

## Abstract

Primosome protein PriB is a single-stranded DNA-binding protein that serves as an accessory factor for PriA helicase-catalyzed origin-independent reinitiation of DNA replication in bacteria. A recent report describes the identification of a novel PriB protein in *Klebsiella pneumoniae* that is significantly shorter than most sequenced PriB homologs. The *K. pneumoniae* PriB protein is proposed to comprise 55 amino acid residues, in contrast to *E. coli* PriB which comprises 104 amino acid residues and has a length that is typical of most sequenced PriB homologs. Here, we report results of a sequence analysis that suggests that the *priB* gene of *K. pneumoniae* encodes a 104-amino acid PriB protein, akin to its *E. coli* counterpart. Furthermore, we have cloned the *K. pneumoniae priB* gene and purified the 104-amino acid *K. pneumoniae* PriB protein. Gel filtration experiments reveal that the *K. pneumoniae* PriB protein is a dimer, and equilibrium DNA binding experiments demonstrate that *K. pneumoniae* PriB's single-stranded DNA-binding activity is similar to that of *E. coli* PriB. These results indicate that the PriB homolog of *K. pneumoniae* is similar in structure and in function to that of *E. coli*.

## Introduction

The survival of cellular organisms depends on complete and faithful duplication of their genetic material. Throughout the life of a cell, the process of DNA replication is challenged by environmental and chemical factors that can bring about damage to the DNA, which can disrupt the DNA replication machinery (replisome) [1]. Since failure to replicate the genome can result in cell death, microorganisms have adapted to these challenges by developing various mechanisms to recognize and repair DNA damage and ensure complete replication of their genetic information [2], [3]. In bacteria, DNA replication restart pathways facilitate reactivation of replisomes that have been disrupted following encounters with DNA damage [4].

*E. coli* has proved to be an excellent model organism to investigate bacterial DNA replication restart pathways. In *E. coli*, DNA replication restart is catalyzed by primosome proteins, including PriA, PriB, PriC, DnaT, and DnaG, that collectively facilitate reloading of the replisome to allow DNA replication to continue [4]. The function of these primosome proteins involves coordinated protein and nucleic acid binding within a large nucleoprotein complex called the DNA replication restart primosome. PriA helicase is the initiator protein that binds to a repaired DNA replication fork and unwinds double-stranded DNA at the fork to produce a short tract of single-stranded DNA (ssDNA) [5], [6], [7]. PriB binds to PriA, stabilizes PriA on the DNA, and stimulates its helicase-activity [6], [8]. The PriA:PriB:DNA ternary complex recruits DnaT to the DNA, which could lead to release of ssDNA from PriB [6], [9]. The replicative helicase, DnaB/C, is recruited to the fork where it unwinds the parental duplex DNA to stimulate priming by DnaG and reloading of the replicative polymerase.

Although studies using *E. coli* have revealed much about the mechanism of DNA replication restart, it cannot necessarily be uniformly applied to all prokaryotes because some organisms do not encode the full complement of primosome protein genes. Genome sequencing projects have revealed that *priA* genes are highly conserved among sequenced bacterial genomes, but *priB*, *priC*, and *dnaT* genes are not. The absence of one or more of these primosome genes from bacterial genomes suggests that there might be mechanistic differences in DNA replication restart pathways across diverse bacterial species.

In accordance with this hypothesis, Hsieh and Huang recently reported the identification of a novel PriB protein in *Klebsiella pneumoniae* [10]. According to their study, the *K. pneumoniae* PriB protein is only 55 amino acids in length, which is considerably shorter than *E. coli* PriB. The sequence of *K. pneumoniae* PriB that appears to be missing is analogous to the amino terminal region of *E. coli* PriB and includes amino acid residues important for dimerization and DNA binding. The authors also report that PriB proteins from *Pectobacterium carotovorum*, *Yersinia ruckeri*, and *Salmonella enterica* have considerably shorter amino acid sequences compared to *E. coli* PriB. The implications are that the PriB homologs from these bacterial species must be different in structure and in function from the well-studied *E. coli* PriB [10].

Here, we report that the PriB protein of *K. pneumoniae* is a full-length PriB homolog whose sequence is the same length as *E. coli* PriB. Our sequence analysis of the other bacterial PriB proteins that have been proposed to be missing amino-terminal sequences reveals that they, too, are full-length PriB homologs whose lengths are comparable to *E. coli* PriB. Furthermore, we have cloned the full-length *priB* gene from *K. pneumoniae*, overexpressed and purified the recombinant *K. pneumoniae* PriB protein, and examined its quaternary structure and DNA binding activity. Our results indicate that the structure and function of *K. pneumoniae* PriB are highly similar to that of *E. coli* PriB. Thus, *K. pneumoniae* PriB does not likely represent a novel PriB homolog.

## Results and Discussion

### Sequence analysis

**Figure 1:**
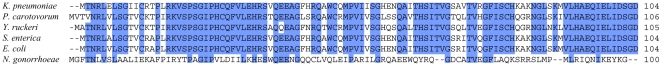
Multiple amino acid sequence alignment of PriB homologs. Amino acid sequences of *Klebsiella pneumoniae* PriB (GenBank ID:YP_001338213), *Pectobacterium carotovorum* PriB (GenBank ID:C6DE14), *Yersinia ruckeri* PriB (GenBank ID:ZP_04617249), *Salmonella enterica* PriB (GenBank ID:AAL23212), *Escherichia coli* PriB (GenBank ID:NP_418622), and *Neisseria gonorrhoeae* PriB (GenBank ID:YP_207725) were aligned using the program ClustalX [15]. Amino acid residues that are identical in at least five of the six aligned PriB proteins are highlighted in blue.

According to the genetic sequence database at the National Center for Biotechnical Information (NCBI), *K. pneumoniae* PriB protein (GenBank ID:YP_001338213) consists of 55 amino acids as predicted *ab initio* by Genemark 2.0. Given that the vast majority of PriB proteins have a sequence of approximately 104 amino acids, we found it striking that *K. pneumoniae* PriB would be shorter to such a significant degree. Therefore, we examined the genome of *K. pneumoniae* in the region upstream of the annotated *priB* gene and noticed that the start codon of the *priB* gene reported in the database is preceded by sequence that codes for a stretch of amino acids that is highly similar to the amino-terminal region of *E. coli* PriB. By including this additional upstream sequence, along with the annotated *K. pneumoniae priB* sequence, we were able to identify an open reading frame in the *K. pneumoniae* genome that codes for a 104-amino acid protein whose amino acid sequence is 95% identical to that of *E. coli* PriB (Figure 1). Since the 55-amino acid *K. pneumoniae* PriB sequence in the NCBI database was predicted *ab initio*, we think it is likely that the *ab initio* gene search incorrectly assigned an internal ATG as the *priB* start codon, resulting in a truncated PriB amino acid sequence being reported in the NCBI database. This truncated PriB sequence appears to have formed the basis for the study by Hsieh and Huang. We propose that the actual amino acid sequence of *K. pneumoniae* PriB is 104-amino acids in length and is highly similar to that of *E. coli* PriB.

We also examined the sequences of the other PriB homologs reported by Hsieh and Huang to be shorter than would be expected based on the sequence of a typical PriB homolog [10]. We found that the amino acid sequence of *Pectobacterium carotovorum* PriB reported in the NCBI database is 106 amino acids in length, and the amino acid sequence of *Salmonella enterica* PriB is 104 amino acids in length. The amino acid sequence of *Yersinia ruckeri* PriB, as reported in the NCBI database, is 55 amino acids in length. Therefore, we analyzed the genome of *Yersinia ruckeri* in the region upstream of the *priB* gene in the same manner as we did for *K. pneumoniae priB* and found additional sequence upstream of the annotated *priB* start codon that codes for the missing amino-terminal region of *Y. ruckeri* PriB. Based on this sequence analysis, we propose that the *priB* genes of *K. pneumoniae*, *P. carotovorum*, *Y. ruckeri*, and *S. enterica* all encode proteins of comparable length to *E. coli* PriB (Figure 1).

### Quaternary structure of *K*. *pneumoniae* PriB

A previous report by Hsieh and Huang describes a 55-amino acid variant of *K. pneumoniae* PriB as a monomeric protein [10], while *E. coli* PriB exists as a homodimer [11], [12], [13], [14]. In *E. coli*, the dimerization interface of PriB is extensive and involves a large number of contacts between individual monomers. Among the interactions are hydrogen bonds that form between the amino-terminal β1 strand of one monomer and the amino-terminal β1 strand of the other monomer [12], [13], [14]. Since these β strands include amino acids 1-11, it is possible that a variant of PriB that lacks a portion of its amino-terminus could exist as a monomeric protein. This appears to be the case for the 55-amino acid variant of *K. pneumoniae* PriB that is missing residues analogous to amino acid residues 1–49 of *E. coli* PriB [10].

**Figure 2:**
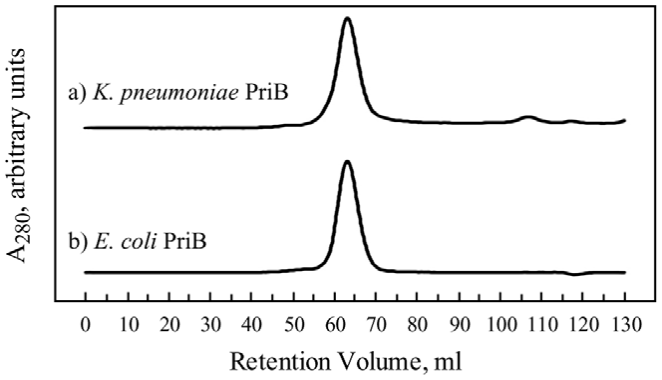
Gel filtration of PriB proteins from *K. pneumoniae* and *E. coli.* Equivalent amounts of (a) *K. pneumoniae* PriB, and (b) *E. coli* PriB, each at approximately 3.4 g/l, were individually resolved through a sephacryl S-100 size-exclusion chromatography column under identical experimental conditions as described in Materials and Methods.

Since our sequence analysis of *K. pneumoniae* PriB suggests that it is a 104-amino acid protein whose sequence is highly similar to that of *E. coli* PriB, we hypothesized that the full-length, 104-amino acid *K. pneumoniae* PriB should exist as a homodimer. To test this hypothesis, we purified the recombinant *E. coli* PriB and *K. pneumoniae* PriB proteins and compared their quaternary structures using gel filtration chromatography. *E. coli* PriB and *K. pneumoniae* PriB each migrate through a sephacryl S-100 size-exclusion chromatography column as a single peak with retention volumes of 62.61 ml and 62.86 ml, respectively (Figure 2). Based on a calibration of the column using proteins of known molecular weight, we determined that *E. coli* PriB migrates as a dimer with a molecular weight of approximately 22.5 kDa and *K. pneumoniae* PriB migrates as a dimer with a molecular weight of approximately 22.4 kDa. These results indicate that *E. coli* PriB and *K. pneumoniae* PriB have highly similar quaternary structures under these experimental conditions.

### DNA binding activity of *K*. *pneumoniae* PriB

Due to the high degree of similarity between *K. pneumoniae* PriB and *E. coli* PriB at the level of primary and quaternary structure, we hypothesized that the mechanism of ssDNA binding is similar between the two PriB homologs. To test this hypothesis, we used fluorescence polarization spectroscopy to measure the DNA binding activity of *K. pneumoniae* PriB to compare it with that of *E. coli* PriB. For these experiments, we measured the apparent dissociation constant for the interaction between *K. pneumoniae* PriB and fluorescein-labeled ssDNA oligonucleotides. The fluorescein tag on the ssDNAs allows us to measure PriB binding to the ssDNA due to the increase in fluorescence anisotropy of the PriB:ssDNA complex relative to the unbound ssDNA. *K. pneumoniae* PriB protein was serially diluted and incubated with 1 nM fluorescein-labeled ssDNA and the fluorescence anisotropy was measured. Apparent dissociation constants were obtained by determining the concentration of PriB needed to achieve 50% binding to each of the various ssDNA substrates.

**Figure 3:**
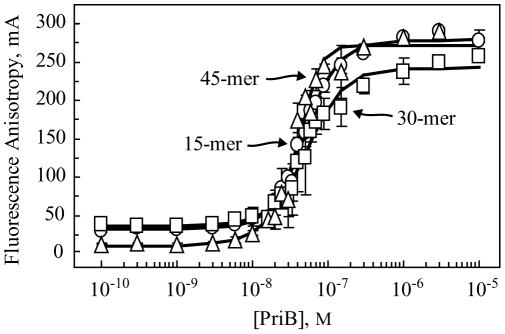
Single-stranded DNA-binding activity of *K. pneumoniae* PriB. PriB protein was diluted serially and incubated with fluorescein-labeled 15-base (circles), 30-base (squares), or 45-base (triangles) ssDNA oligonucleotides as described in Materials and Methods. Measurements are reported in triplicate and error bars represent one standard deviation of the mean.

When *K. pneumoniae* PriB was incubated with each of the fluorescein-labeled ssDNA oligonucleotides, we observed a PriB-dependent increase in fluorescence anisotropy, indicating that *K. pneumoniae* PriB binds to the ssDNAs (Figure 3). The apparent dissociation constants for 15-base, 30-base, and 45-base fluorescein-labeled ssDNAs are 50±3 nM, 45±7 nM, and 62±14 nM, respectively. As a comparison, *E. coli* PriB's apparent dissociation constant for the same 30-base fluorescein-labeled ssDNA, measured using the same instrument and under similar experimental conditions, is 34.6±7.7 nM [12]. These results indicate that the affinity of *K. pneumoniae* PriB for ssDNA is highly similar to that of *E. coli* PriB.

Overall, the results of our study support the hypothesis that *K. pneumoniae* PriB is a 104-amino acid ssDNA-binding protein whose structure and function mirrors that of *E. coli* PriB. Therefore,*K. pneumoniae* PriB does not likely represent a novel PriB homolog.

## Materials and Methods

### Cloning K. pneumoniae priB and E. coli priB

The *priB* gene of *K*. *pneumoniae* was amplified from strain MGH78578 genomic DNA by polymerase chain reaction (PCR) using primers oML292 (5′-GCG TAT TCC ATA TGA CCA ACC GTC TGG AGC TG) and oML293 (5′-GTC ACG GAT CCC TAG TCT CCA GAA TCT ATC AAT TC). The PCR-amplified product was cloned into the pET28b expression vector (Novagen) using NdeI and BamHI restriction sites. The resulting plasmid contains a six-Histidine tag upstream of the complete coding sequence of the *K. pneumoniae priB* gene, which is under the control of a T7 promoter for overexpression in hosts harboring T7 polymerase controlled by the lacUV5 promoter. The cloning of the *priB* gene of *E. coli* was described previously [12]. The fidelity of the *priB* genes was confirmed by DNA sequencing. All plasmids were individually transformed into BL21(DE3) *E. coli* to allow recombinant protein overexpression following induction with isopropyl-β-D-thiogalactopyranoside (IPTG).

### Purification of *K*. *pneumoniae* PriB and *E. coli* PriB

*K. pneumoniae* PriB protein was purified from BL21(DE3) *E. coli* harboring the pET28b:Kpn-*priB* plasmid. Cells were grown in Luria-Bertani medium containing 50 µg/ml kanamycin and 50 µg/ml chloramphenicol at 37°C until an OD_600_ of 0.4 was reached. Expression of PriB was induced with 0.5 mM IPTG for 3 hr and cells were harvested by centrifugation at 5,000 × g. Cells were lysed in 10 mM Tris–HCl pH 8, 10% (v/v) glycerol, 0.5 M NaCl, 10 mM imidazole, 1 mM β-mercaptoethanol, 1 mM phenylmethylsulfonyl fluoride (PMSF) by sonication on ice. The lysate was clarified by centrifugation at 40,000 × g. His-tagged PriB was bound to nickel-NTA agarose (Qiagen) and eluted in 10 mM Tris–HCl pH 8, 10% (v/v) glycerol, 0.5 M NaCl, 250 mM imidazole, 1 mM β-mercaptoethanol. The nickel-NTA agarose eluate was dialyzed against 10 mM Tris–HCl pH 8, 10% (v/v) glycerol, 0.3 M NaCl, 1 mM β-mercaptoethanol, concentrated, and resolved through a HiPrep HR 16/10 sephacryl S-100 size-exclusion chromatography column (GE Healthcare) in 10 mM Tris–HCl pH 8, 10% (v/v) glycerol, 0.5 M NaCl, 1 mM β-mercaptoethanol. PriB fractions were pooled, concentrated, and stored at −80°C.

*E. coli* PriB protein was purified from BL21(DE3) *E. coli* harboring the pET28b:Ec-*priB* plasmid. Cells were grown in LB medium containing 50 µg/ml kanamycin and 50 µg/ml chloramphenicol at 37°C until an OD_600_ of 0.4 was reached. Expression of PriB was induced with 1 mM IPTG for 3 hr and cells were harvested by centrifugation at 5,000 × g. Cells were lysed in 10 mM Hepes pH 7, 10% (v/v) glycerol, 1 M NaCl, 10 mM imidazole, 0.1 M glucose, 1 mM β-mercaptoethanol, 1 mM PMSF by sonication on ice. The lysate was clarified by centrifugation at 40,000 × g. His-tagged PriB was bound to nickel-NTA agarose (Qiagen) and eluted in 10 mM Hepes pH 7, 10% (v/v) glycerol, 1 M NaCl, 250 mM imidazole, 1 mM β-mercaptoethanol. The nickel-NTA agarose eluate was concentrated and purified through a HiPrep HR 16/10 sephacryl S-100 size-exclusion chromatography column (GE Healthcare) in 10 mM Hepes pH 7, 10% (v/v) glycerol, 1 M NaCl, 1 mM β-mercaptoethanol. PriB fractions were pooled, concentrated, and stored at −80°C.

### Gel filtration

Purified *K. pneumoniae* PriB and *E. coli* PriB were individually applied to a HiPrep HR 16/10 sephacryl S-100 size-exclusion chromatography column (GE Healthcare) and resolved at 0.35 ml/min in 10 mM Tris–HCl pH 8, 10% (v/v) glycerol, 0.5 M NaCl, 1 mM β-mercaptoethanol. The column was calibrated under identical conditions with protein standards of known molecular weight: thyroglobulin (670,000 Da), bovine gamma-globulin (158,000 Da), chicken ovalbumin (44,000 Da), equine myoglobin (17,000 Da), and vitamin B12 (1,350 Da) (BioRad). Protein was detected in the column eluate by measuring the absorbance at 280 nm.

### Equilibrium DNA binding assays

Fluorescence polarization spectroscopy was performed at 25°C with a Beacon 2000 fluorescence polarization system (Invitrogen). PriB proteins were diluted serially from 10,000 nM to 0.01 nM into 20 mM Tris–HCl pH 8, 50 mM NaCl, 4% (v/v) glycerol, 1 mM MgCl_2_, 1 mM β-mercaptoethanol, 0.1 mg/ml bovine serum albumin (BSA) and incubated with 1 nM 3′-fluorescein-labeled ssDNA oligonucleotides of varying lengths: 15-mer (5′-TAG CAA TGT AAT CGT), 30-mer (5′-GCG TGG GTA ATT GTG CTT CAA TGG ACT GAC), 45-mer (5′-GCC GTG ATC ACC AAT GCA GAT TGA CGA ACC TTT GCT CCA GTA ACC) in a total volume of 100 µl. Apparent dissociation constants (K_d,app_) were calculated by determining the concentration of PriB required to bind 50% of the fluorescein-labeled ssDNA. The unbound state is reported by the fluorescence anisotropy of the fluorescein-labeled ssDNA in the absence of PriB. The fully-bound state is reported by the fluorescence anisotropy of the fluorescein-labeled ssDNA in the presence of a sufficient concentration of PriB to saturate the fluorescence anisotropy signal. Data are reported in triplicate and associated uncertainties are one standard deviation of the mean.
